# Aggressive T-cell Lymphoma Smoldering As Hemophagocytic Lymphohistiocytosis: A Diagnostic and Medical Challenge

**DOI:** 10.7759/cureus.76757

**Published:** 2025-01-01

**Authors:** Christina Said, Karim Bitar, Fadia Elias

**Affiliations:** 1 Medicine and Medical Sciences, University of Balamand, Beirut, LBN; 2 Internal Medicine and Clinical Immunology, Mount Lebanon Hospital University Medical Center, Beirut, LBN; 3 Hematology/Oncology, Mount Lebanon Hospital University Medical Center, Beirut, LBN

**Keywords:** ebv, familial hemophagocytic lymphohistiocytosis, hemophagocytic lymphohistiocytosis (hlh), macrophage activation syndrome (mas), pancytopenia, peripheral t-cell lymphoma, secondary hemophagocytic lymphohistiocytosis (shlh)

## Abstract

Hemophagocytic lymphohistiocytosis (HLH) is an aggressive hematologic disease based on widespread immune activation and tissue destruction. This uncommon condition can be primary as well as secondary to infection or malignancy. However, HLH diagnosis rarely contributes to unveiling an underlying malignancy. The most crucial prognostic factor of HLH is timely diagnosis and treatment. However, due to the rarity of this syndrome and the variable clinical presentations, HLH is often underdiagnosed. A 12-year-old boy complained of recurrent fever and fatigue for the past four months. He presented with pancytopenia, hyperferritinemia, hypertriglyceridemia, elevated liver enzymes, and a severe inflammatory profile. The diagnosis of HLH disease was established. During further evaluation, a right subaxillary palpable lymph node was found. The biopsy revealed a peripheral T-cell lymphoma, not otherwise specified (PTCL, NOS). The bone marrow was also infiltrated with evidence of dissemination to the skeleton on a positron emission tomography (PET) scan (stage IV). The patient was treated with six cycles of CHOEP (cyclophosphamide, doxorubicin, oncovin, etoposide, prednisone) protocol but relapsed and passed away three months later. This case report sheds light on the importance of early recognition and treatment of HLH, as well as searching for underlying malignancy or disease, even in young patients.

## Introduction

Hemophagocytic lymphohistiocytosis (HLН) is a life-threatening hematological disorder characterized by an uncontrolled activation of cytotoxic T-cells and macrophages, resulting in severe inflammation. It is an extremely rare condition with a suspected incidence of around 1-2 per million [1], likely reflecting the underdiagnosis of this disease. Classically, HLH has been classified as a primary (inherited) or secondary (acquired) condition. Primary HLH is a familial condition (FHL) that typically presents at a younger age and usually suggests a congenital immunodeficiency syndrome or an inherited genetic defect. On the other hand, secondary HLH more frequently occurs in adults and has been associated with acquired infections such as Ebstein Barr virus (EBV) and cytomegalovirus (CMV), malignancies such as lymphomas or other immunological conditions such as systemic lupus erythematous. The clinical presentation of HLH typically includes a prolonged high-grade fever and multiorgan failure, with splenic, hepatic, respiratory, renal, and neurological involvement [2]. However, the rarity of this disease limits treatment options, and the prognosis remains poor, with a reported five-year survival rate of 54% in children [3].

In this report, we will discuss the case of a young male who was diagnosed with HLH after a long period of fever of unknown origin. This established diagnosis elicited further workup that led to the discovery of an underlying rare subtype of T-cell lymphoma. By highlighting the clinical presentation and management of these rare entities, we intend to shed light on these conditions in order to improve their early recognition and benefit patients’ outcomes.

## Case presentation

A 12-year-old boy complained of fever and fatigue for the past four months. He had already been hospitalized twice in another hospital for the same clinical picture and had been diagnosed with pancytopenia secondary to a viral infection (white blood cells: 1,900/mm3, hemoglobin: 8.9 g/dl, platelets: 50,000/mm3). However, no serological tests were done and the patient’s condition did not improve. The patient was previously healthy with no personal or family history and no recent vaccinations or medications intake. His vital signs were normal except for a fever of 38.5 ℃. On physical exam at presentation, the patient was very pale, with a palpable right axillary lymph node and hepatosplenomegaly.

Laboratory tests showed pancytopenia (white blood cells: 1,100/mm3, hemoglobin: 8.1 g/dl with MCV: 85, platelets: 44,000/mm3) with an elevated ferritin (2,000 ng/ml), triglycerides (387 mg/dl), aspartate transaminase (AST) (190 IU/l), gamma glutamyl transpeptidase (GGT) (121 IU/l), alkaline phosphatase (ALP) (636 IU/l), c-reactive protein (CRP) (388 mg/l) and erythrocyte sedimentation rate (ESR) (72 mm/h), with a low fibrinogen level (1 g/l). On further evaluation, the patient was found to have an elevated reticulocyte count, lactate dehydrogenase (LDH) and indirect bilirubin; however, haptoglobin level was normal. Moreover, direct and indirect coombs tests were negative and schistocytes were absent from the peripheral blood smear. The patient had a nonhemolytic normocytic anemia.

In order to assess for secondary infectious causes, viral serologies were done. The EBV PCR and serology turned out positive for anti-viral capsid antigen IgM and viral capsid antigen IgG, while other serologies (CMV, herpes simplex virus, human immunodeficiency virus, hepatitis C virus, hepatitis B virus, coxsackie, parvovirus B19, human T-lymphotropic virus, human herpes virus 6 and 8) were negative. All cultures (hemocultures, urine culture, stool culture) were also negative. An auto-immune panel (antinuclear antibody, anti-DNA, C3, C4, c-antineutrophil cytoplasmic antibody, p-antineutrophil cytoplasmic antibody, antiphospholipid antibodies, lupus anticoagulant, and anti-cardiolipin antibodies) was done to rule out macrophage activation syndrome (MAS).

**Figure 1:**
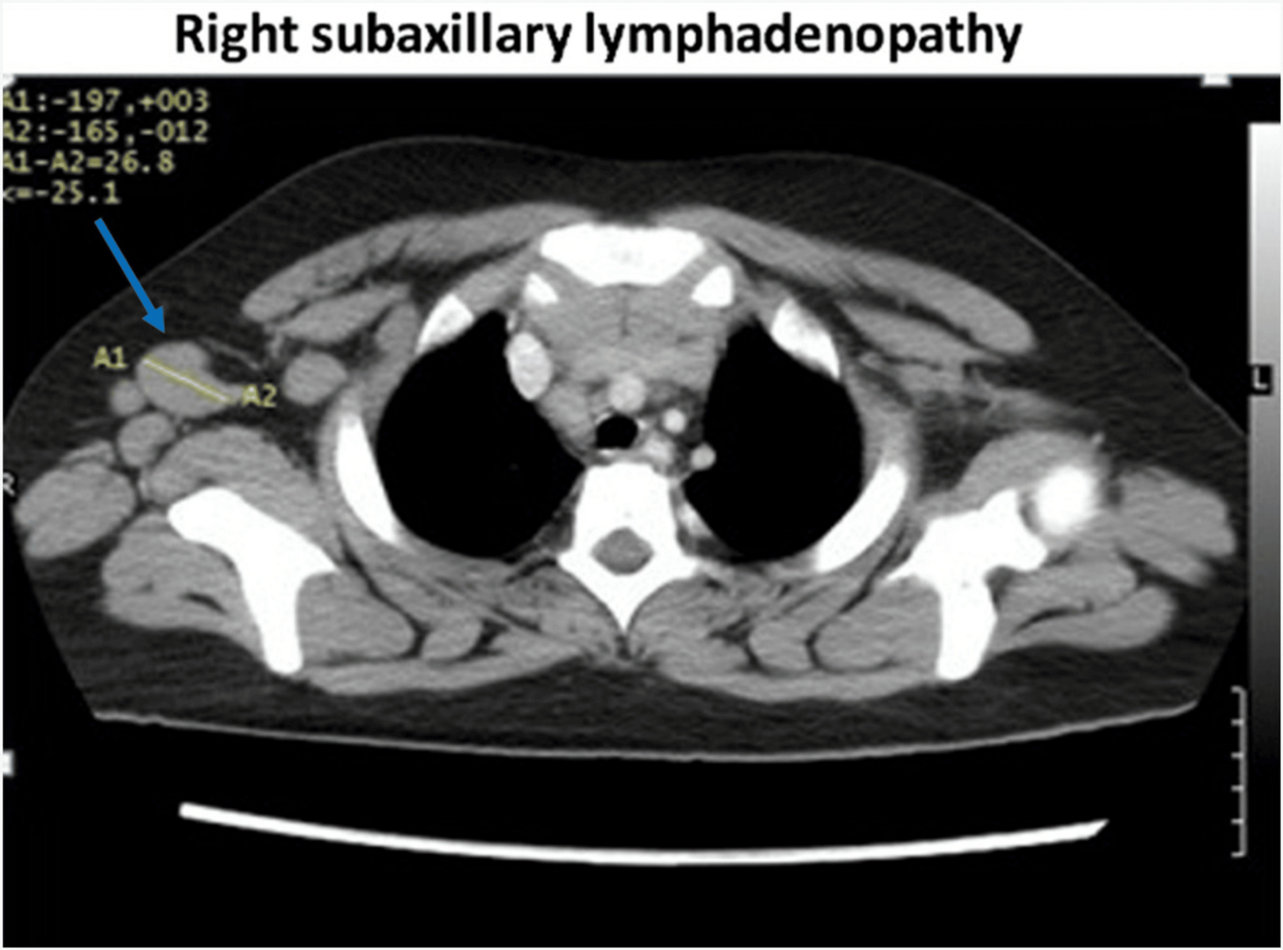
Abdominal CT scan showing the right subaxillary lymph node mass (blue arrow)

A thoraco-abdomino-pelvic CT scan with IV contrast showed right axillary (3 cm), mediastinal and abdominal lymphadenopathies with hepatosplenomegaly (Figure 1). Thus, based on the HLH-2004 protocol, five out of eight criteria were met, and the diagnosis of HLH syndrome was established. With the absence of specific rheumatological symptoms and the auto-immune panel turning out negative, the diagnosis of HLH was confirmed.

**Figure 2:**
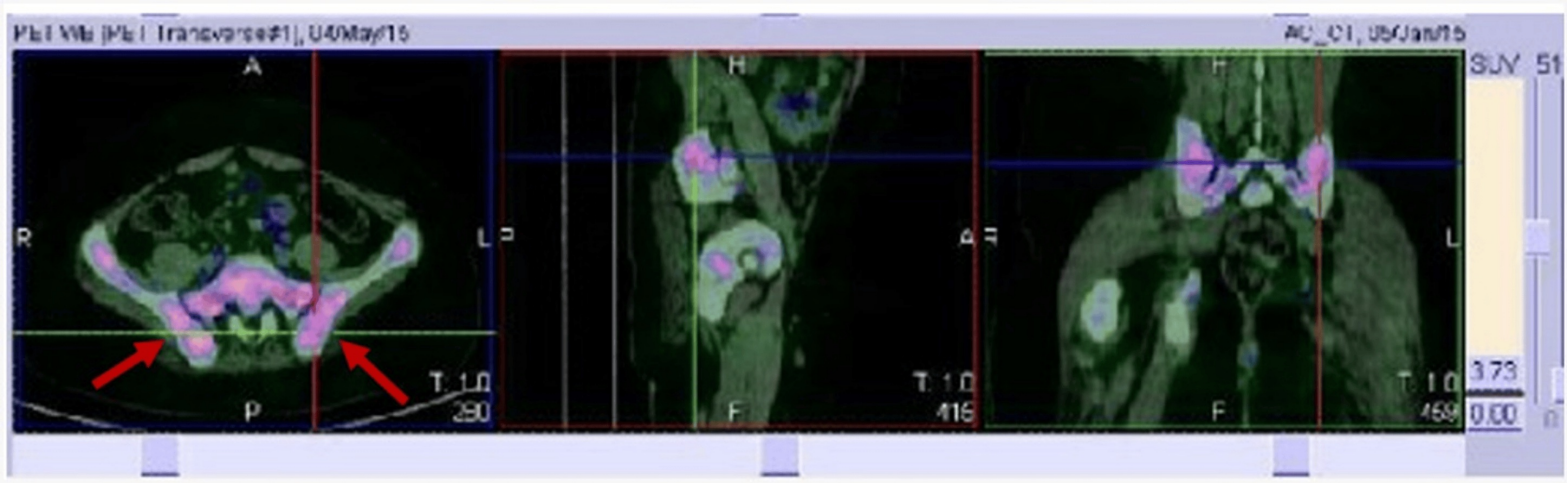
PET scan showing bilateral iliac bone metastases (red arrows)

A positron emission tomography (PET) scan was positive for bone metastases in the ribs and iliac bones (Figure 2).

Biopsies of the right axillary lymph node and bone marrow were performed and revealed a peripheral T-cell lymphoma, not otherwise specified (PTCL, NOS). A bone marrow aspiration showed hypocellularity with atypical cells of large round nuclei and blue cytoplasm (22%) but without a picture of hemophagocytosis. Flow cytometry revealed B/T inversion with no evidence of acute leukemia.

The patient was treated with blood and platelet transfusions, hydration, allopurinol, alkalinization, antipyretics, and corticosteroids. After the pathological result of the biopsies, he received six cycles of chemotherapy according to the CHOEP protocol (cyclophosphamide, doxorubicin, oncovin, etoposide, prednisone). The patient improved clinically, and HLH parameters normalized. Three months later, he relapsed and died before receiving any second-line treatment.

## Discussion

**Figure 3:**
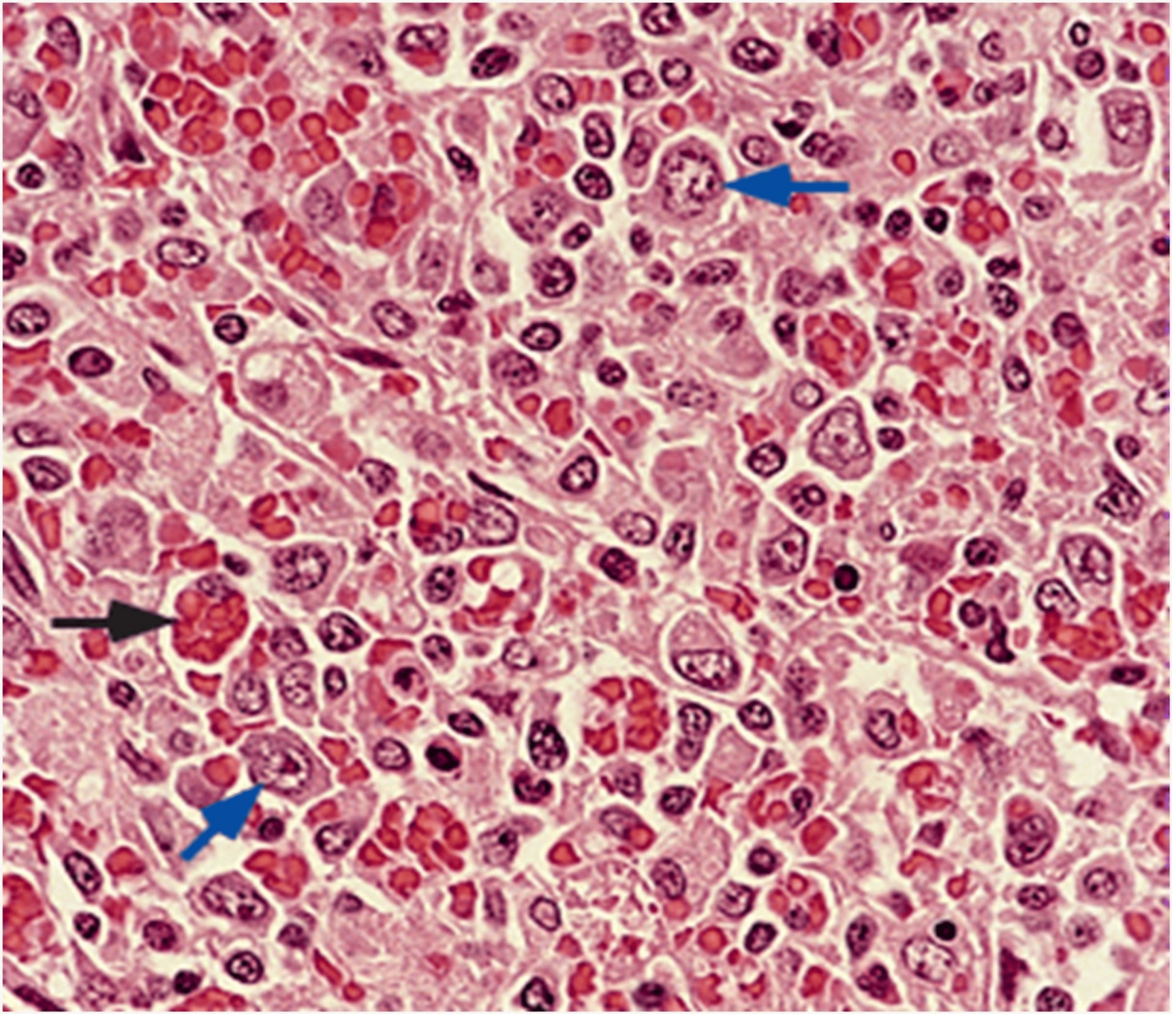
Large T cell lymphoma with reactive hemophagocytosis (black arrow) Source: Warnke et al. [5]

HLH is a condition of excessive inflammation and tissue destruction caused by abnormal downregulation of activated macrophages and cytotoxic lymphocytes. In this uncontrolled immune state, macrophages become excessively activated, and natural killer (NK) cells and cytotoxic lymphocytes fail to complete their normal function and eliminate these macrophages. This dysregulated activation leads to the secretion of an abnormally high number of cytokines and interferon-gamma [4]. The resulting destructive cytokine storm is the core reason for the multiorgan involvement and high mortality rate of this disease. In addition, hemophagocytosis can also be seen in bone marrow biopsy or biopsies of immune tissues (spleen, liver, lymph nodes) as a sign of macrophage overactivation. It is characterized by the presence of red blood cells, platelets, or white blood cells engulfed in the cytoplasm of macrophages (Figure 3). However, this criterion is neither specific nor required for the diagnosis of HLH, and in our case, it was not present in the bone marrow nor in the lymph node biopsies.

FHL mainly manifests in childhood or with several recurrent episodes of HLH during a patient's lifetime. It is an autosomal recessive disease, and the reported mutations affect the perforin pathways of cytotoxic T and NK cells [6]. In fact, many FHL loci have been identified, and patients can be tested for them. However, our young patient did not undergo any genetic study, and he did not report any similar familial cases. On the other hand, secondary HLH is often associated with viral infections such as EBV, CMV, parvovirus, and less commonly, gram-negative bacteria, parasites, and fungi [7]. Indeed, our patient tested positive for EBV infection, which was found to be one of the most common viral etiologies associated with HLH [8]. Recently, it has been suspected that COVID-19 vaccination has been linked with an increased likelihood of cytokine storms resulting in HLH [9]. In our case, there was no history of recent vaccination.

HLH has also been reported to be associated with malignancies, most commonly B or T lymphoid cancers, leukemias, and solid tumors. Our patient had an uncommon type of lymphoma: Peripheral T-cell lymphoma (PTCL), which accounts for 5% of all lymphomas [10]. Among T-cell lymphomas (TCL), peripheral T-cell lymphoma not otherwise specified (PTCL, NOS) is one of the most frequent subtypes associated with HLH (21% of all TCL-HLH cases) [11]. This type of lymphoma is different from all other types of lymphomas and does not fit into any other category. It is a rapidly growing type of lymphoma that affects mostly older patients. In contrast, our patient was a very young 12-year-old patient. PTCL, NOS, often involves the lymph nodes, but widespread disease is not infrequent, as in our case, and is associated with a high risk of disease recurrence. Newly identified genomic characteristics in this rare disease play an important role in the prognosis and treatment [12]. Since lymphoma has often been a hidden trigger of HLH, the use of positron emission tomography-guided imaging in patients diagnosed with HLH has been found to be extremely useful in locating the source of the disease [13].

In some cases, patients with malignancies develop HLH following an acute viral infection that triggered the disease. In fact, it is assumed that our patient was unknowingly immunocompromised due to his PTCL diagnosis and caught an EBV infection that progressed into HLH. It has been found that when HLH is associated with a malignancy, it becomes more life-threatening than the malignancy itself, and the prognosis is very poor for any malignancy-associated HLH [14]. That is why very early recognition and diagnosis of HLH in cancer patients is crucial, and prompt intervention could be life-saving.

HLH's initial presentation is not usually specific, and this could lead to a late fatal diagnosis, similar to our case study. The patient with HLH typically presents with an acute febrile illness, and in some cases, seizures, altered mental status, skin rashes, hepatitis, acute respiratory distress syndrome (ARDS), and renal failure may be reported. The definite diagnosis relies on the HLH-2004 guidelines where five out of eight criteria are required (Table 1), including fever, cytopenias, splenomegaly; hypertriglyceridemia and/or hypofibrinogenemia, ferritin ≥500 ng/mL, elevated soluble interleukin 2 receptor, hemophagocytosis in bone marrow or spleen or lymph nodes, and low or absent NK cell activity [2]. Our patient met five of these criteria as he did not have hemophagocytosis in the bone marrow biopsy, and levels of soluble interleukin-2 (IL-2) receptor and NK cell activity were not measured. Moreover, HScore is another tool/protocol used to diagnose HLH. However, comparative studies have shown that both HLH-2004 and HScore have good diagnostic accuracy and can be used interchangeably [15].

**Table 1:** HLH-2004 diagnostic criteria HLH - hemophagocytic lymphohistiocytosis

Diagnosis established if any five criteria are fulfilled
Fever >38.5°C for >7 days
Splenomegaly
Cytopenias (affecting > 2 out of 3 lineages): Hemoglobin < 9g/L, Platelets < 100x10^9^/L, Absolute neutrophil count <1.0x10^9^/L
Hypertriglyceridemia (fasting triglycerides > 265 mg/dL) and/or hypofibrinogenemia (fibrinogen < 1.5 g/L)
Ferritin >500 mg/L
Soluble CD25 (IL-2 receptor) >2,400 U/mL
Low or no NK cell activity
Hemophagocytosis in the bone marrow or spleen or lymph node

An important differential diagnosis is macrophage activation syndrome (MAS), which is a form of HLH associated with an underlying rheumatologic disease, most commonly systemic juvenile idiopathic arthritis. The differentiation between these two conditions, which is based on clinical symptoms and auto-immune panel, is crucial due to different treatment approaches [16].

Treatment relies mainly on suppressing the life-threatening immune activation. Usually, FHL is treated with hematopoietic stem cell transplantation, while in secondary HLH, the focus is on treating the underlying cause. The lymphoma-associated hemophagocytic syndrome is treated similarly to lymphomas with the CHOP protocol (cyclophosphamide, doxorubicin, oncovin, prednisone) and the addition of etoposide. As in the case of our patient, the PTCL lymphoma was treated with CHOP protocol with the addition of etoposide to target the HLH.

Acutely ill patients could be treated with the HLH-2004 protocol, which includes administration of etoposide and dexamethasone [17]. Etoposide acts as an inhibitor of topoisomerase II, leading to the breakage of double-stranded DNA. Thus, it can specifically consume the activated T lymphocytes and inhibit the production of inﬂammatory cytokines [18]. In fact, etoposide has played a monumental role in decreasing the mortality of lymphoma-associated HLH. A previous study showed that patients receiving etoposide-based therapeutic regimens had a lower rate of early mortality than those who did not receive etoposide-containing treatment [19].

Despite its contributions, the present study does have some limitations. Firstly, systematic data on lymphoma-associated HLH are scarce, limiting comparative studies. Secondly, genetic studies were not performed on this young patient to rule out an underlying FHL. In addition, the detection of sCD25 and NK cell activity was also not tested in this study.

## Conclusions

This case illustrates the importance of early recognition of HLH presentation in young patients as well as the necessity for a thorough work-up to search for underlying causes. It highlights the importance of maintaining a high index of suspicion for hematological malignancies in the differential diagnosis of HLH, regardless of the patient's age. Several aspects of the clinical presentation of HLH contribute to its underdiagnosis, including the rarity of the syndrome, the variable clinical presentation, and the lack of specificity of the clinical and laboratory findings. However, delay in the HLH diagnosis is often the greatest barrier to prompt treatment and successful outcomes.
